# Identification of critical versus robust processing unit operations determining the physical and biochemical properties of cassava‐based semolina (gari)

**DOI:** 10.1111/ijfs.14857

**Published:** 2020-11-08

**Authors:** Andrés Escobar, Eric Rondet, Layal Dahdouh, Julien Ricci, Noël Akissoé, Dominique Dufour, Thierry Tran, Bernard Cuq, Michèle Delalonde

**Affiliations:** ^1^ The Alliance of Bioversity International and the International Center for Tropical Agriculture (CIAT) CGIAR Research Program on Roots Tubers and Bananas (RTB) Apartado Aéreo 6713 Cali Colombia; ^2^ Qualisud University of Montpellier CIRAD SupAgro University of Avignon University of La Réunion 73 rue JF Breton Montpellier 34398 France; ^3^ CIRAD, UMR Qualisud, F‐34398 Montpellier France; ^4^ Faculty of Agronomical Sciences University of Abomey Calavi Cotonou Benin; ^5^ UMR IATE, CIRAD, INRA University of Montpellier Montpellier SupAgro, Montpellier France

**Keywords:** Cassava, gari, process optimisation, process scale, product quality, quality attributes

## Abstract

The gari‐making process involves several unit operations (U.O.), some of which strongly influence the quality of the end product. Two contrasting process scales (laboratory‐scale *vs* conventional) were compared in order to identify which U.O. were affected by the change of scale. U.O. that changed end‐product characteristics depending on process scale were deemed *critical*; whereas U.O. that resulted in similar characteristics were deemed *robust*. The classification depended on quality attributes considered: rasping and roasting were *critical* for physical properties, in particular particle size which ranged from 0.44 to 0.89 mm between the two process scales; and *robust* for biochemical properties. In contrast, fermentation and pressing were *critical* for biochemical properties such as lactic acid content (0.93–1.88 g/100 g dry matter after pressing), which influences the perception of flavour, and *robust* for physical properties. This classification between critical and robust operations help quality control of gari, by pinpointing which U.O. control specific quality characteristics.

## Graphical abstract

By comparing two process scales for elaboration of gari, we evaluated which unit operations are sensitive to process scale, and hence critical in determining the quality of the end‐product, and which operations result in similar end‐product quality independently of process scale.

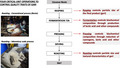

## Introduction

In West Africa, cassava is widely consumed after processing into different types of staple foods, such as gari (Bechoff *et al*., 2016, Oyeyinka et al., 2019). Gari is a granulated foodstuff with many regional variations depending on consumer preferences and final consumption mode (Adinsi et al., 2019). Processing conditions therefore also vary depending on the region, in order to adjust physico‐chemical characteristics and end‐product quality according to local expectations. In addition, to increase shelf life and avoid health risks, the moisture content and cyanogens content of gari must be maintained below defined thresholds (Njankouo Ndam et al., 2019). To meet these technological, sanitary and sensorial requirements, gari processors combine and adjust the parameters of several unit operations: peeling, washing and rasping the raw cassava roots; and fermenting, pressing, sieving and roasting the pulp to produce gari (Akingbala et al., 2005). Several studies have evaluated the impact of unit operations and cassava genotypes on the quality of gari (Agbor‐Egbe & Lape Mbome, 2006, Nwancho et al., 2014, Olaoye et al., 2015), in particular fermentation (Moorthy & Mathew, 1998, Iwuoha & Eke, 1996, Sokari & Karibo, 1996, Kostinek et al., 2005, Montagnac et al., 2009) and roasting (Chuzel et al., 1995a, Igbeka et al., 1992, Sobowale et al., 2017, Ezeocha et al., 2019, Samuel et al., 2010). More recently, Escobar *et al*. (2018) investigated the combined effect of the successive unit operations on the final quality of gari, under controlled laboratory conditions. This approach led to a comprehensive description of the evolution of biochemical, physical and hydro‐textural properties of cassava along the gari production steps.

Comparing these studies is delicate, however, as different methods were used to characterise different sets of quality attributes. To further understand how unit operations influence the quality of gari, in this work a single methodological framework (Escobar et al., 2018) was applied to characterise gari processing at two contrasting scales (conventional process by small‐scale processors at village level *vs*. a smaller‐scale process using laboratory equipment). The main objective was to identify which unit operations are affected by processing scale and hence are critical in determining the quality of the end product, as opposed to robust unit operations, which are not affected by processing scale. Specific objectives were as follows: (i) to describe the physical and biochemical mechanisms at play during each unit operation; (ii) to evaluate how robust or critical the main unit operations for gari are; and (iii) to assess their impact on product characteristics. Identifying robust or critical operations has practical applications when developing equipment to mechanise the gari process while maintaining end‐product quality according to consumer expectations.

## Materials and methods

### Raw materials and locations for processing

Gari production with the conventional process was conducted in Benin, using roots of a local cassava cultivar named Odoungbo purchased from a local farmer, and processed by a small‐scale cassava processor in the Plateau department. Gari production with the laboratory‐scale process was conducted in Colombia, using roots from the genotype MCOL‐22 grown, harvested and processed at CIAT headquarters in Palmira, Valle del Cauca Dept., Colombia.

### Production of gari from cassava roots

**Figure 1:**
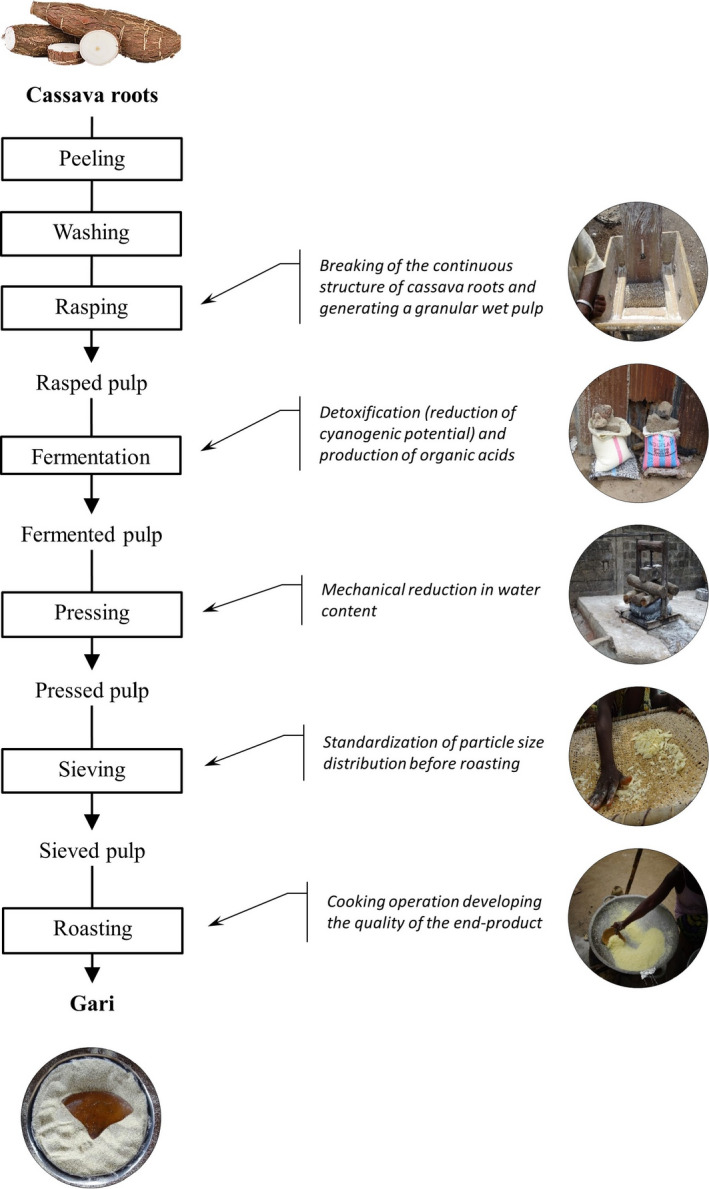
Process diagram to transform cassava roots into gari.

Cassava roots were transformed into gari (Fig. 1) according to the two process scales.

#### Conventional process

After peeling and washing, 100 kg of cassava roots was rasped using a motorised mechanical rasper made of a perforated cylindrical sheet metal (diameter of the rotor 190 mm; diameter of the holes 5 mm, surface occupied by the holes 55% of the cylinder surface) rotating at 256 ***g*** (1550 r.p.m.). The rasped pulp was placed in woven plastic bags and pressed to remove water. The bags were left under pressure in the press for 72 h under ambient conditions (35 °C during daytime, 25 °C during night‐time, 85% relative humidity) for lactic acid fermentation. The press was composed of a manually operated mechanical screw. To extract as much liquid as possible, increasing pressure was applied four times: The initial pressure application at time t_0_, then three successive increases at 10, 12 and 17 min after *t*_0_. The fermented cake was sieved using a 3.35 mm mesh sieve. The resulting pulp was roasted over a wood fire using a metallic pan of surface area 0.63 m^2^ and average temperature 184 °C. Sieved pulp (3 kg) was gradually introduced into the pan (4.8 kg pulp m^−2^) while stirring: at the beginning, after 2 or 3 min and after 5 or 6 min. The tool for stirring was a triangular piece of gourd shell the operator moved in circles and sometimes along figures of eight, at the same time tossing and turning the product. The total roasting time was 8 min. At the end of roasting, the gari was collected, cooled and stored in air‐tight plastic containers until characterisation.

#### Laboratory‐scale process

Gari was produced at laboratory‐scale according to Escobar *et al*. (2018). Cassava roots (3 kg) were rasped using a disc rasper rotating at 22 g (438 rpm, disc diameter 203 mm, diameter of the holes 5 mm, surface occupied by the holes 40% of the disc surface; Skymsen PA‐7SE, Bateas, Brazil). Cassava pulp was fermented for 72 h in a pressurised (0.04 bar) thermostatic fermentation unit (35 °C and 55% relative humidity). The fermented pulp (300 g) was pressed in a compression unit (IFTS, Foulayronnes, France) for 1 h at 3 bar and then manually sieved using a 3.35 mm mesh sieve. Sieved pulp (90 g) was sprinkled onto a 0.69 m^2^ hot plate (128 °C ± 8 °C; 0.26 kg pulp m^−2^) and cooked for 3 min while stirring with a metal spatula, using circular movements and tossing and turning the product. A second portion of sieved pulp (90 g) was then added and cooked for 3 more minutes, also under stirring. The total roasting time was 6 min.

### Pulp and gari characterisation

#### Water content

The water content, *w* (g g^−1^) of the cassava roots, pulp and gari was determined according to Eq. 1, where $m_{w}$ is the mass of water evaporated during oven drying (105 °C until constant mass) and $m_{s}$ the mass of solid after drying.

(1) $$w=\frac{m_{w}}{m_{s}}$$

#### Solid true mass density of dried cassava roots

The solid true mass density $\rho_{s}^{*}$ (kg m^−3^) of dried and milled roots was measured using a helium multivolume pycnometer 1305 Micromeritics (Quantachrome Instruments, Odelzhausen, Germany). Analyses were performed in triplicate. The solid true mass density of the dried roots was 1565 (±2) kg m^−3^.

#### *Solid volume fraction of the fresh cassava root*s *and gari*

Total volume (*V*) and solid volume fraction ($\theta_{s}$) of fresh cassava roots and gari were calculated based on their mass measured in air (*W_a_*) and immersed in liquid (*W_l_*)‐‐water and paraffin for fresh roots and gari, respectively (Archimedes’ principle). Calculations are detailed by Equation 2 and Equation 3 where $\rho_{l}^{*}$ is the mass density of the liquid (1.00 and 0.88 g cm^−3^ for water and paraffin, respectively), and *W_a_* and *W_l_* the masses of fresh roots or gari measured in air and in liquid, respectively. Analyses were performed in triplicate.

(2) $$V=\frac{W_{a}‐W_{l}}{\rho_{l}^{*}}$$

(3) $$\theta_{S}=\frac{V_{s}}{V}=\frac{\rho_{l}^{*}}{\rho_{S}^{*}}\times \frac{W_{a}}{\left(W_{a}‐W_{l}\right)\times \left(1+w\right)}$$

#### Solid volume fraction of pulp, fermented pulp and pressed pulp

The solid volume fraction ($\theta_{s}$) of pulp, fermented pulp and pressed pulp was determined (Eqn 4) either by sample coring using cylindrical cores of known volume *V* (19 cm^3^ for laboratory‐scale process and 244 cm^3^ for conventional process) or by using directly the known volume *V* of the compression unit in which pulp was pressed (laboratory‐scale process). The mass of dry solids *m_s_* in the sample of volume *V* was determined by drying, from which the volume of the solids *V_s_* was calculated. Measurements were performed in triplicate.

(4) $$\theta_{s}=\frac{V_{s}}{V}=\frac{m_{s}}{\rho_{s}^{*}.V}$$

#### Particle size distribution

The particle size distribution of rasped pulp (300 g) was measured by wet sieving according to (Da et al., 2013). The particle size distribution of sieved pulp (100 g) and of gari was determined by conventional dry sieving using a set of seven sieves (Test Sieve, Fisher Scientific Co., Portsmouth, USA) with a decreasing mesh size (3.35, 2, 1.4, 0.85, 0.6, 0.3 and 0.15 mm). The particle size distribution was described by the median particle diameter (*d_50_*). All the analyses were performed at least in triplicate.

#### Damaged cell ratio

**Figure 2:**
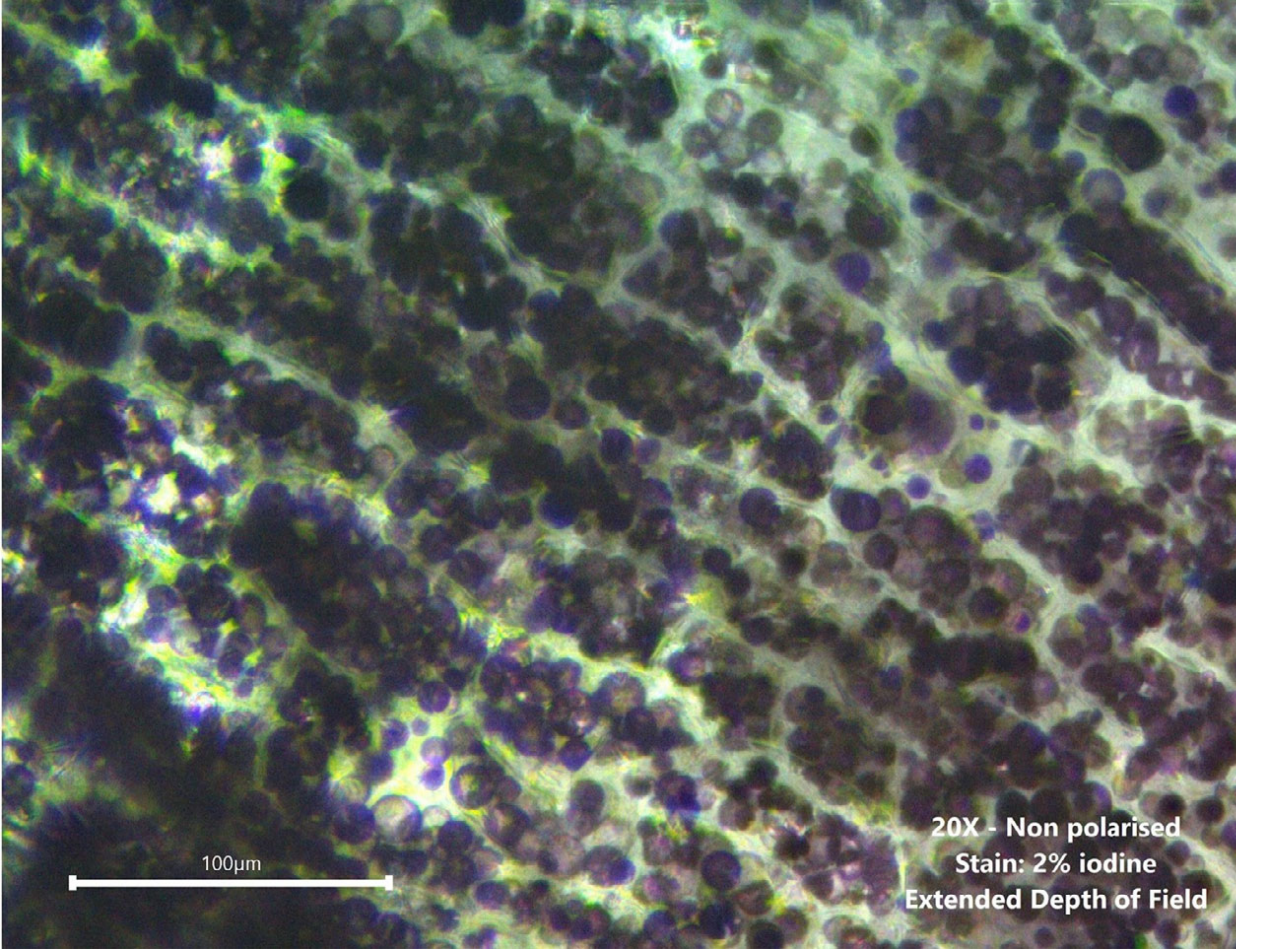
Micrograph of cassava root tissue showing starch granules contained in intracellular compartments. Starch granules are stained in dark blue by iodine solution (2%).

During rasping, damaged cells release starch granules from intracellular compartments (Fig. 2) to the extracellular space. An original method developed in this study estimated the damaged cell ratio in cassava pulp (rasped, fermented, pressed) based on the evaluation of the distribution of starch content between the extracellular and intracellular compartments. The pulp sample (200 g) was washed on a 53 µm mesh sieve until the washing water was clear, to remove extracellular starch from the pulp. The remaining intracellular starch content was measured by enzymatic method (Holm et al., 1986). The damaged cell ratio was estimated using Eqn 5, where *m_is_* is the dry mass of intracellular starch and *m_p_,* the dry mass of total starch in the sample studied.

(5) $$Damagedcellsratio\left(\text{\textbackslash{}\%}\;\right)=100‐\left(\frac{m_{\mathrm{is}}}{m_{p}}\right)\times 100$$

#### Cyanogenic potential and titratable acidity

Cyanogenic potential (HCN, µg HCN g^−1^ dry matter) and titratable acidity (g of lactic acid/100 g of dry matter) were quantified on rasped, fermented, pressed pulp and gari using, respectively, the colorimetric procedure (Sánchez et al., 2009, Essers et al., 1993)) and an automatic Titroline apparatus (Schott Schweiz AG, St. Gallen, Switzerland) (Escobar et al., 2018). All the analyses were performed in duplicate.

#### Soluble sugars and lactic acid contents

Sugars and lactic acid contents were quantified by HPLC (Agilent Technologies Software Inc. 2006^©^) (Holloway et al., 1989, Sánchez et al., 2013) using 0.5 g of dry milled samples of rasped, fermented, pressed pulp and gari (Escobar et al., 2018). All the analyses were performed in triplicate.

#### Degree of starch gelatinisation of garis

Gelatinisation enthalpies of sieved pulp and gari (respectively, ΔH_pulp_ and ΔH_gari_, J g^−1^ of dry starch) were measured by differential scanning calorimetry (DSC 8500 Perkin Elmer, Norwalk, USA) (Escobar et al., 2018). Starch gelatinisation (%) was calculated using Eqn 6.

(6) $$\text{Starch gelatinisation}\;\left(\text{\textbackslash{}\%}\;\right)=\frac{\left(\Delta \text{Hpulp}\;‐\Delta \text{Hgari}\right)}{\Delta \text{Hpulp}}\times 100$$

##### Swelling capacity of gari

The swelling capacity of gari (mL water mL^−1^ gari) was estimated in triplicate by measuring the volume of a gari sample (10 mL) before and after dispersion in 40 mL of water (Olayinka Sanni, 1994). Experiments were carried out in triplicate.

##### Colour of gari

Gari colour was determined in triplicate according to the L*a*b* colour notation system using a Minolta Chroma Meter (CR‐400 Konica Minolta, Japan).

##### Statistical analysis

The statistical significance of measured values was verified using single factor analysis of variance (ANOVA) and Tukey test on replicated data. Statistical analyses were carried out with JMP software (SAS Institute, Cary, NC, USA) at 95% confidence level.

## Results and discussion

### Physical characteristics

#### Hydro‐textural characteristics

**Figure 3:**
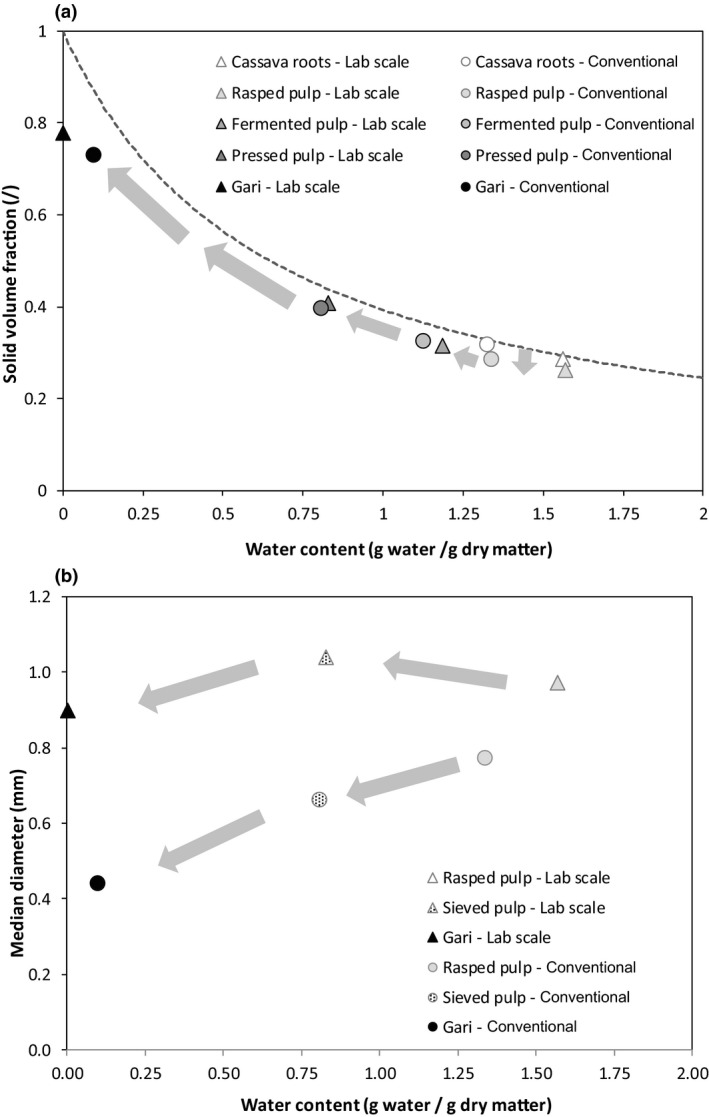
Hydro‐textural diagram showing the evolution of solid volume fraction (a) and median diameter (b) vs. water content during gari processing by conventional process (○) and laboratory‐scale process (Δ).

The experimental values of water content and solid volume fraction of native cassava roots, pulp and gari for the two process scales were plotted on the hydro‐textural diagram (Fig. 3a) (Ruiz et al., 2005). The area below the saturation curve (dotted line, Eqn 7) corresponds to unsaturated samples in which water does not fill all the spaces between the fragments of rasped vegetal tissues (spaces between the fragments and/or intercellular spaces within fragments).

(7) $$\theta \left(w_{\mathrm{sat}}\right)=\frac{1}{1+\frac{\rho_{s}^{*}}{\rho_{w}^{*}}w_{\mathrm{sat}}}$$

Fresh cassava roots were located on the saturation curve, which confirmed that roots are a continuous cellular matrix saturated with aqueous liquid. Although the initial water contents of the two cassava genotypes were significantly different (Table 1), their solid volume fraction and saturation degree were similar (Fig. 3a). The rasping operation did not significantly change the water content, but reduced slightly the solid volume fraction (rasped pulp, Table 1 and Fig. 3a). The main effect of rasping was to modify the distribution of the liquid phase due to cell damage, as a large fraction of water initially located inside the cells was redistributed towards the extracellular and intercellular spaces (Rondet et al., 2019).

**Table 1:** Physical characteristics of fresh cassava roots, pulp (rasped, fermented, pressed, sieved) and gari, produced by conventional and laboratory‐scale processes

	Water content (g g^−1^ dry matter)	Solid volume fraction (/)	Median diameter d_50_ (mm)	Damaged cells ratio (%)
Conventional process				
Cassava roots	1.32 (±0.01)^b^	0.32 (±0.01)^ab^	n/a	n/a
Rasped pulp	1.34 (±0.06)^b^	0.28	0.77	66
Fermented pulp	1.13 (±0.02)^d^	0.32 (±0.02)^ab^	n/d	67
Pressed & sieved pulp	0.81 (±0.03)^e^	0.40 (±0.06)^a^	0.66	63
Gari	0.10 (±0.02)^f^	0.73	0.44	n/d
Laboratory‐scale process				
Cassava roots	1.56 (±0.02)^a^	0.29 (±0.01)^b^	n/a	n/a
Rasped pulp	1.57 (±0.01)^a^	0.26 (±0.01)^b^	0.97 (±0.01)^b^	63 (±4)^a^
Fermented pulp	1.19 (±0.01)^c^	0.32 (±0.01)^ab^	n/d	65 (±2)^a^
Pressed & sieved pulp	0.83 (±0.01)^e^	0.41	1.04 (±0.01)^a^	68 (±4)^a^
Gari	0.002 (±0.01)^g^	0.78	0.89 (±0.01)^c^	n/d

n/a, not applicable; n/d, not determined.

Standard deviations were calculated from triplicate samples. In each column, values with a different letter are significantly different (*P* < 0.05). In some cases, the quantity of sample available was too small to carry out replicates, so that standard deviations and significant differences could not be evaluated.

Both the fermentation and pressing operations reduced water content and increased the solid volume fraction, thus maintaining the pulp in a near‐saturated state (Fig. 3a). Drying, draining and gravitational flow mechanisms (due to the own weight of the sample) were at play to reduce water content during fermentation, while draining was the main phenomenon during pressing. Both operations reduced the differences between the two cassava cultivars, even though operating conditions were markedly different between conventional and laboratory processes (Table 1).

Roasting caused the largest reduction in water content through evaporation, from 0.8 to 0.1 g g^−1^ dry matter or less (Table 1), and increased the solid volume fraction of gari particles up to 0.73–0.78, independently of the process scale. The final water content of gari was low enough to ensure preservation at ambient temperature, with water activity in the range 0.3–0.5. This highlighted the strong influence of roasting conditions on the final characteristics of gari, in this case related to the lower load of pulp in the roasting pan at laboratory‐scale, resulting in more heating and water evaporation.

#### Particles size characteristics

The initial rasping operation broke the native continuous cellular structure of cassava roots and generated discrete particles. Due to the higher rasping rotation speed, the median diameter of these particles was significantly lower for the conventional compared to the laboratory process (0.77 vs. 0.97 mm, Table 1). The fermentation, pressing and sieving operations modified the median diameter, with a slight increase in the case of the laboratory‐scale process and decrease in the case of the conventional process (Fig. 3b). The roasting operation led to significant differences between the two process scales: At laboratory‐scale, the particle size of gari was close to that of the pulp (0.90 mm) whereas at conventional scale, the final particle size was half that of the pulp (0.44 mm) (Fig. 3b). These differences may be related to the evaporation rate of water, which controls the extent of various phenomena during roasting such as agglomeration, shrinkage and starch gelatinisation.

#### Damaged cell ratio

The rasping operation damaged the cells markedly (63–66%, Table 1), with no significant difference in the damaged cells ratio between conventional and laboratory‐scale processes. The absence of difference between the two process scales also indicates that the cultivar had no effect on the physical characteristics of the products obtained. During the following fermentation and pressing operations, neither mechanical forces nor bacterial activity did cause further changes in damaged cell ratio, contrary to other observations (Williams et al., 2019, Ha et al., 2020; Table 1). This may indicate that the maximum extent of cell damage was reached after rasping.

### Biochemical and functional characteristics

#### Cyanogenic potential

During fermentation, cyanogenic potential decreased significantly and in similar proportions whatever the process conditions (Table 2), as previously observed (Agbor‐Egbe & Lape Mbome, 2006 , Montagnac et al., 2009 ). The usual explanation is that during rasping and subsequent fermentation, linamarase‐containing vacuoles are ruptured, resulting in the hydrolysis of cyanogenic glycoproteins and evaporation of cyanide under ambient conditions. Pressing also significantly reduced the cyanogenic potential of the pulp, whatever the process scale, thanks to the draining of cyanogenic soluble compounds with water (Onabolu et al., 2002). Roasting further reduced cyanogenic potential (Table 2), due to the volatilisation of HCN (O'Brien et al., 1992). Laboratory‐scale roasting reduced cyanogenic potential more than the conventional process (respectively, 6.3 and 12.8 µg HCN g^−1^ dry matter, after roasting), possibly because of the lower load of the roasting pan at laboratory‐scale. Nevertheless, at both process scales, cyanogenic potential remained above the FAO recommended value of 2 µg g^−1^ dry matter (FAO/WHO, 1995), which constitutes a health concern due to long‐term exposure to cyanide.

**Table 2:** Biochemical characteristics (cyanogenic potential, titratable acidity, lactic acid content) of pulp and gari, produced by conventional and laboratory‐scale processes

	Cyanogenic potential (µg HCN/g dry matter)	Titratable acidity (g lactic acid/100 g dry matter)	L‐lactic acid (g/100 g dry matter)
Conventional process			
Rasped pulp	248.5 (±0.4)^b^	0.695 (±0.008)^e^	1.54 (±0.022)^d^
Fermented pulp	106.8 (±0.1)^d^	1.279 (±0.002)^a^	2.75 (±0.025)^a^
Pressed pulp	74.4 (±0.1)^f^	1.080 (±0.011)^c^	1.88 (±0.046)^b^
Gari	12.8 (±0.1)^g^	1.125 (±0.010)^b^	1.67 (±0.023)^c^
Laboratory‐scale process			
Rasped pulp	292.3 (±1.9)^a^	0.590 (±0.001)^f^	0.36 (±0.010)^g^
Fermented pulp	117.1 (±0.1)^c^	0.676 (±0.001)^e^	1.84 (±0.017)^b^
Pressed pulp	81.2 (±0.1)^e^	0.559 (±0.001)^g^	0.93 (±0.013)^f^
Gari	6.3 (±0.1)^h^	0.769 (±0.001)^d^	1.42 (±0.042)^e^

Standard deviations were calculated from duplicate samples. In each column, values with a different letter are significantly different (*P* < 0.05).

#### Titratable acidity and lactic acid

Titratable acidity and lactic acid content were significantly higher in the rasped pulp used in the conventional process (Table 2), reflecting differences between the two cassava genotypes. Lactic fermentation predictably increased titratable acidity and lactic acid content, while pressing had the opposite effect due to drainage of acidic compounds with water. The final roasting operation significantly increased titratable acidity for both process scales, and lactic acid content only in the case of the laboratory‐scale process (Table 2), due to water evaporation and subsequent concentration of the chemical compounds remaining in gari particles. Lactic acid content and titratable acidity followed the same general pattern across unit operations, but were not strongly correlated.

#### Sugar contents

The two cassava cultivars in this study had contrasting sugar contents (Table 3). The cultivar used for the laboratory‐scale process had a high total sugars content (42.8 mg g^−1^ dry matter), with high sucrose and low fructose, whereas the cultivar used for the conventional process had a higher fructose content. For both processes, fermentation consumed the sugars initially present in the rasped pulp, to levels below the quantification limit of the analytical method. After fermentation, sugars remained below detectable levels in the pulp and final gari.

**Table 3:** Sugar contents of rasped pulp produced by conventional and laboratory‐scale processes

	Sugar contents (mg g^−1^ dry matter)	
	Conventional process	Laboratory‐scale process
Sucrose	1.7 (±0.02)^b^	37.8 (±0.3)^a^
Glucose	< L.Q.	4.3 (±0.52)^a^
Fructose	10.9 (± 0.2)^a^	0.6 (± 0.08)^b^
Total sugars	12.6 (± 0.2)^b^	42.8 (± 0.3)^a^

Standard deviations were calculated from triplicate samples.

Value with a different letter in the same line is significantly different (*P* < 0.05). L.Q.: Limit of Quantification.

#### Functional characteristics

Starch was almost fully gelatinised in gari samples during roasting, with a slightly higher degree of gelatinisation for the laboratory‐scale process (92.4%, Table 4). The degrees of starch gelatinisation were consistent with values reported previously (>65%) in gari (Chuzel et al., 1995b). The degree of starch gelatinisation controls the ability of gari to absorb water before human consumption and therefore contributes to swelling capacity and to the final texture of the product. The small difference in degree of starch gelatinisation between conventional and laboratory‐scale processes may explain the lack of significant difference in swelling ability (Table 4). The gari produced at laboratory‐scale was significantly darker (L* = 77.4) with more yellow (b* = 27.0) and red (a* = 7.3) hues compared to gari from the conventional process (Table 4), possibly due to harsher roasting conditions leading to stronger Maillard reactions. Maillard reactions are classically observed during heat treatment of partly dried products and produce specific colour and aromatic compounds that contribute to the sensory characteristics (colour and taste) of the end product.

**Table 4:** Functional characteristics of gari produced by conventional and laboratory‐scale processes

	Functional characteristics of gari	
	Conventional process	Laboratory‐scale process
Degree of starch gelatinisation (%)	89.4 (±0.2)^a^	92.4 (±0.3)^b^
Swelling capacity (mL water mL^−1^ gari)	3.1 (±0.2)^a^	3.1 (±0.1)^a^
Colour ‐ Lightness (L*)	91.4 (± 0.3)^a^	77.4 (± 1.0)^b^
Colour ‐ Redness (a*)	‐0.27 (± 0.03)^b^	7.3 (± 0.39)^a^
Colour ‐ Yellowness (b*)	10.7 (± 0.2)^b^	27.0 (± 0.9)^a^

Standard deviations were calculated from triplicate samples.

Values with a different letter in the same line are significantly different (*P* < 0.05).

### Critical operations determining macroscopic and biochemical characteristics of gari

The rasping operation was critical in determining the particle size (median diameter) of the rasped pulp and gari at different processing scales, but did not influence the hydro‐textural characteristics, which remained similar to the fresh roots. This is in line with previous findings that rasping conditions (number and diameter of holes of the metal sheet, rotation speed), combined with the properties of cassava roots, in particular dry matter content and hardness, determine the particle size distribution of the rasped material (Escobar et al., 2018). Regarding biochemical composition, the rasping operation was robust with no influence of scale on the quality of the gari end product.

The fermentation operation was considered robust for physical characteristics, as it reduced the hydro‐textural differences between pulp samples rasped at different processing scales and did not modify particle size, thus giving similar saturated structure to fermented pulp. On the other hand, fermentation was considered critical for biochemical composition, because lactic bacteria produced lactic acid to different extents at different processing scales, which may play a role in the perception of flavour of the final gari product (Moorthy & Mathew, 1998, Kostinek et al., 2005, Montagnac et al., 2009, Iwuoha & Eke, 1996, Sokari & Karibo, 1996). Compared to the changes during fermentation, initial genotypic differences in the biochemical composition of the fresh roots were small, which explains that unit operations before fermentation had little or no influence on biochemical properties of the end product.

Similarly, the pressing operation was considered robust as it did not modify particle size and also reduced the hydro‐textural differences between pulp samples from the two processing scales. This result was interpreted as due to complete draining of all the mechanically extractable liquid, leaving the pressed pulp with similar water contents (0.81 and 0.83 g g^−1^ dry matter, Table 1) and in the same hydro‐textural state, as suggested by the concept of maximum dryness (Ruiz et al., 2007). On the other hand, pressing was considered critical for biochemical composition, as drainage reduced lactic acid, titratable acidity and cyanogenic compounds to different levels at different processing scales, depending on the initial quantities present before pressing, as well as the type of press. Other studies also reported the role of pressing in reducing biochemical compounds (Akingbala *et al*., 1991; Taiwo *et al*., 2001; Onabolu *et al*., 2002 ).

Sieving the pressed pulp generated discrete particles, but with only small changes in median diameters compared to the rasped pulp (Table 1). Two opposite mechanisms could simultaneously occur during the pressing and sieving operations (Escobar et al., 2018): (i) an increase in particle diameter due to wet agglomeration of tissue fragments and (ii) a decrease in particle diameter due to drainage of intracellular and extracellular liquid. The specific contribution of these opposite mechanisms seems to depend on the process type. In this study, wet agglomeration was favoured in the laboratory‐scale process and drainage of liquid in the conventional process. Hence, sieving can be considered critical in determining particle size of the gari end product; however, its effect was smaller than that of the roasting operation. Other studies also identified the influence of sieving on particle size (Sanni *et al*., 2008; Ezeocha *et al*., 2019).

The roasting operation was critical in determining hydro‐textural characteristics and particle size of gari at different processing scales, as a result of the combined effects of agglomeration and shrinkage mechanisms, driven mainly by water evaporation. Liquid capillary bridges between particles at the beginning of roasting induce agglomeration as the departure of water reduces the length of the bridges, until free liquid is exhausted. Simultaneously, dry matter content and viscosity increase in the capillary bridges, inducing a shift from liquid‐like to a more solid‐like behaviour. This phenomenon is enhanced by starch granule gelatinisation in the capillary bridges, which contributes to increasing viscosity. Shrinkage occurs in parallel to agglomeration, as water evaporation reduces the volume of pulp particles. Shrinkage lasts as long as water content is high enough to maintain strain capacity, in other words as long as the pulp remains in the rubbery state, above the glass transition (Perdomo et al., 2009). The agglomeration and shrinkage mechanisms were not sufficient to compensate the volume of evaporated water, so that some internal porosity appeared in the final gari, as indicated by their location below the saturation curve on the hydro‐textural diagram (Fig. 3). These various phenomena determine the texture of the end product and depend on the evolution of the quantity of free water remaining in the product; water content at the beginning and during roasting therefore plays a critical role on the quality of the final product. Thus, for particle size, both the rasping and roasting operations combined together in influencing the quality of the gari end product (Sobowale et al., 2017, Ezeocha et al., 2019). For biochemical and functional characteristics, roasting was critical in determining the colour and degree of starch gelatinisation of the final gari (Chuzel et al., 1995b), but was considered robust for titratable acidity and lactic acid content, as these parameters were influenced more by the fermentation and pressing operations.

## Conclusions

The comparison of two process scales for elaboration of gari allowed identifying which unit operations are sensitive to process scale and hence critical in determining the quality of the end product, and which operations result in similar end‐product quality independently of process scale (referred to as robust operations). Among unit operations for gari processing, the classification between critical and robust depended on the quality properties considered. Rasping and roasting were critical for physical properties, in particular particle size, which in the end determine the texture of the final product, and were robust for biochemical properties. Roasting was also critical in determining the colour of the end product. In contrast, fermentation and pressing were critical for biochemical properties, which influence the perception of flavour and the safety (low cyanogens content) of the end product, and were robust for physical properties. In addition to process parameters, the cultivar played a role in determining biochemical properties, but not physical properties. These results contribute to solving quality issues during gari processing, by pinpointing which unit operations are more likely to be the source of specific problems. When modifying the process, for instance upgrading a piece of equipment or introducing a new technology, these findings on the critical or robust nature of unit operations also assist in anticipating and addressing potential impacts on product quality, thereby ensuring that the final product still meets consumers’ expectations.

## Author Contribution

**Andrès Escobar:** Conceptualization (equal); Data curation (lead); Formal analysis (equal); Investigation (lead); Methodology (equal); Writing‐original draft (equal); Writing‐review & editing (equal). **Eric Rondet:** Data curation (equal); Investigation (equal); Methodology (equal); Supervision (equal); Writing‐original draft (equal); Writing‐review & editing (equal). **Layal Dahdouh:** Data curation (equal); Investigation (equal); Methodology (equal); Supervision (equal); Writing‐original draft (equal); Writing‐review & editing (equal). **Julien Ricci:** Data curation (supporting); Investigation (equal); Methodology (equal); Supervision (supporting). **Noël Akissoe:** Investigation (equal); Methodology (equal); Supervision (equal); Validation (equal); Writing‐original draft (equal). **Dominique Dufour:** Conceptualization (lead); Funding acquisition (lead); Investigation (equal); Methodology (equal); Project administration (equal); Resources (equal); Supervision (equal); Writing‐original draft (supporting); Writing‐review & editing (equal). **Thierry Tran:** Data curation (supporting); Funding acquisition (equal); Investigation (supporting); Methodology (supporting); Project administration (equal); Resources (equal); Supervision (equal); Writing‐original draft (equal); Writing‐review & editing (equal). **Bernard Cuq:** Conceptualization (equal); Investigation (equal); Methodology (equal); Supervision (lead); Writing‐original draft (equal); Writing‐review & editing (equal). **Michele Delalonde:** Conceptualization (equal); Investigation (equal); Methodology (equal); Supervision (equal); Writing‐original draft (equal); Writing‐review & editing (equal).

## Conflict of interest

The authors declare that they have no conflict of interest.

## Ethical Guidelines

Ethics approval was not required for this research.

### Peer review

The peer review history for this article is available at https://publons.com/publon/10.1111/ijfs.14857.

## Data Availability

Data are available on request from the authors.
